# Bifunctionalised acid-base hierarchically structured monolithic microreactor for continuous-flow tandem catalytic process of cyanocinnamate synthesis

**DOI:** 10.1038/s41598-024-77146-7

**Published:** 2024-10-25

**Authors:** Agnieszka Ciemięga, Katarzyna Maresz, Julita Mrowiec-Białoń

**Affiliations:** Institute of Chemical Engineering, Polish Academy of Sciences, Bałtycka 5, Gliwice, 44-100 Poland

**Keywords:** Chemistry, Engineering, Materials science

## Abstract

The results on zirconia-amine bifunctional modification of hierarchically porous silica monoliths for continuous-flow processes ar presented. The study reports the synthesis and properties of the modified porous monoliths and their performance in the tandem process of deacetalization-Knoevenagel condensation reaction. The properties of the materials were studied by thermal analysis, FTIR spectroscopy, XRF and nitrogen adsorption. It was found that both active centres were uniformly distributed along the monolithic cores, and the antagonistic interaction of the acid and base centres was not observed. It was shown that hydrophobicity of the monolith surface has opposite effects on the efficiency of the studied reactions. The overall rate of the tandem process depends on the rate of the condensation reaction. The performance of the bifunctional microreactors was checked and compared with those of cascade-connected monofunctional microreactors and batch reactor. The yield of cyanocinnamate product obtained in both flow systems was ca. 78%; however, pressure drop in the bimodified reactor was only half of that in the cascade. An effective way of water supply to the reaction system has been proposed. The research demonstrates the benefits of using flow-through structured micro-/mesoreactors in tandem reaction processes.

**Supplementary Information:**

The online version contains supplementary material available at 10.1038/s41598-024-77146-7.

## Introduction

Tandem reactions are two or more consecutive reactions that lead to the final product without the addition of new substrates, with no intermediate isolation, and using a fixed work-up procedure. Each subsequent reaction step makes use of the chemicals obtained in the previous one. Thus, it offers a convenient and complete multi-step chemical synthesis of desired products from commercially available precursors^1^. The advantages of this synthesis, i.e. high efficiency and reduction of waste associated with chemical production, meet the requirements of green chemistry and the concept of sustainable development. The tandem method has been adopted for the synthesis of biologically important natural products, agrochemicals and pharmaceuticals, e.g. alkaloids, steroids, lactones, quinazolines, and hormones^2–5^. Although it has been used for both homo- and heterogeneous cascade reactions, the most promising strategies are related to the advantages of supported catalysts, which can be easily separated from the reaction mixture and reused. The fabrication of a bifunctionalised heterogeneous catalyst typically involves multistep modification with active centres of pre-selected supports. Various catalysts have been obtained using nanoparticles, powdered porous supports with well-defined pore structure, i.e. SBA-15, MCM-41, MOFs, zeolites and polymers^6–14^. Tandem reactions usually involve at least two different catalytically active species, and often with antagonistic properties. Several articles reported on the combination of organic groups containing acid-base catalysts such as 4-ethylphenylsulfonic acid/aminopropyl^15^ureidopropyl/3-[2-(2-aminoethylamino)ethyloamino]propyl^16^, 3-mercaptopropyl/aminopropyl^17^. The synthesis of such bifunctional catalysts requires an appropriate design of the system to avoid undesired interactions of the species.

The tandem processes are typically carried out on a laboratory scale in batch mode; however, some structural reactors have also been successfully used for multi-step production of pharmaceutically relevant and fine chemicals. Kirschning et al^18,19^. developed a microreactor consisting of macroporous glass and functionalised polymer for the three-step derivatisation of steroids. Recently, a polymer bifunctional catalyst was proposed for the deacetalization-Knoevenagel condensation reaction carried out in a mild conditions and in flow system^20^. Continuous-flow micro- and mesoreactors are the attractive tools of modern technologies designed to produce new molecules in a greener and more sustainable way in commercial quantity. They have shown to be an efficient, environmentally friendly and less costly alternative for processes performed periodically^21–24^. Unlike batch reactors, they provide efficient mixing¸ safe handling and easier control of process parameters^25,26^. Furthermore, this technology is ready for scale-up without significant modifications and the need for mass and heat transfer optimisation by implementing the numbering-up approach^27^. As a result of these exceptional advantages, the use of microreactors has spread rapidly in the recent decades. They offer facilitated synthesis of chemicals in terms of high yield, selectivity, low energy consumption, low reaction volume, and good stability. The high surface-to-volume ratio also allows for better management of heat and mass transfer.

The aim of this work was to combine the benefits of micro-flow technology and multifunctional heterogeneous catalysis to elaborate an innovative reaction system, based on structured monolithic microreactors, for sequential transformation synthesis. The core of the elaborated microreactor was a monolith with hierarchical pore structure of meso- and macro-pores. The presence of mesopores resulted in a large specific surface area, and thus an ability to host high concentration of catalytic functionalities, while the interconnected network of very large macropores allowed unimpeded flow of fluids through the entire reactor. A tortuous structure of flow-through macropores present in the monoliths boosts mass and heat transport with a positive effect on reaction kinetics. Moreover, the proposed reactor is characterized by dimension of channels attributed to microreactors, i.e. a diameter in the range of 10 to 500 μm, and it has also the features of a mesoreactor, as its length is greater than 500 μm^28^. Silica monoliths has good mechanical, chemical and thermal stability, easy access to internal and external surfaces, and the presence of reactive silanols on the surface that qualifies them as versatile hosts for active centres with different catalytic properties.

Herein, the surface of the monoliths was functionalised with acid-base species, i.e. zirconia and diamine active sites using the post-synthesis method. The bifunctional microreactor was tested in deacetalization-Knoevenagel condensation, considered as a model tandem reaction. The performance of the reactor was compared with that of monofunctional microreactors of the same structure, connected in cascade and with batch process using powdered bifunctional monolith. Two ways of water supply to the flow system have been proposed. To the best of our knowledge this is the first report on a bifunctional continuous-flow microreactor with zirconia and amine acid-base centres for the studied tandem reaction.

## Experimental

### Materials

Polyethylene glycol 35 000, (Sigma-Aldrich), tetraethoxysilane (TEOS, 99%, ABCR), HNO_3_ (65%, Avantor), cetyltrimethylammonium bromide (CTAB, Sigma-Aldrich), ammonia solution (Avantor, 25%), zirconium propoxide (70% in 2-propanol, Sigma-Aldrich), 3-(2-aminoethylamino)propyldimethoxymethylsilane (97%, ABCR) 3-(2-aminoethylamino)propyltrimethoxysilane (97%, ABCR), anhydrous ethanol (Avantor), toluene (99.8%, Avantor), acetonitrile (99%, Chempur), ethyl cyanoacetate (98%, Alfa Aesar), benzaldehyde (99%, Sigma Aldrich), benzaldehyde dimethyl acetal (99%, Alfa Aesar) were applied as received.

## Catalysts preparation

### Monoliths synthesis

Silica monoliths were synthesised according to method described previously^29^. Briefly, polyethylene glycol was dissolved in 1 M nitric acid at room temperature. The mixture was than cooled in the ice bath and TEOS was added dropwise. After 30 min of vigorous stirring CTAB was poured into the flask and the mixing was continued for additional 30 min. The homogeneous sol was transferred to cylindrical shaped polypropylene tubes, sealed, and kept at 40 ^o^C for 8 days. The obtained white rods were washed with distilled water and subjected to hydrothermal treatment in 1 M ammonia solution at 90 ^o^C for 6 h. The monoliths were then washed again, dried at least 3 days at 40 ^o^C, and finally calcined at 550 ^o^C for 8 h.

## Monoliths functionalization

Silica cylindrical monoliths (4-cm length and 0.45 cm in diameter) were soaked in zirconium propoxide solution in ethanol to obtain materials with a 2.3 wt% content of Zr. The samples were placed in a closed vessel, to prevent the solvent evaporation and kept at 70 ^o^C for 24 h. Then they were washed in ethanol by intense shaking, dried at 70 ^o^C and 110 ^o^C and finally calcined at 500 ^o^C for 5 h.

Zr-doped monoliths were embedded in heat-shrinkable PTFE tubes equipped with inlet and outlet connectors to obtain microreactors, and modified with a 3-(2-aminoethylamino)propyldimethoxymethylsilane using two methods. In the first, the modification was carried out by circulating the diamine precursor solution (0.2 mmol/g_silica_) in ethanol (20 ml) through the monolith at 50 ^o^C for 6 h with a flow rate of 0.5 ml/min. Next, it was washed with ethanol and dried at 50 ^o^C for 24 h, then at 110 ^o^C for 24 h. In the second method, the monolith was soaked with the amine precursor solution in the amount corresponding to the total pore volume of this material. The wet sample was sealed and stored at 50 ^o^C. The remaining steps of the procedure followed those applied for the flow method.

## Materials characterization

Nitrogen adsorption/desorption isotherms were obtained in Micromeritics ASAP 2020 analyser at -196 ^o^C. Specific surface area, pore volume and pore size distribution were determined using BET and BJH methods. Scanning electron microscopy (SEM, PhenomPure Thermo Scientific, BSD detector, gold layer 3 nm) was used to study the morphology of the materials. Thermogravimetry was employed to determine the concentration of amines and their distribution along the monolith. The analysis was performed in Mettler Toledo TGA/SDTA 851 device. The samples were heated in the range of 25–800 ^o^C with a heating rate of 10 ^o^C/min and an air flow rate 60 ml/min. The acidic properties of the amine and zirconia modified samples were determined using FTIR spectrometer (Invenio, Bruker) and pyridine as the probe molecule. The sample was pressed into pellets using a hydraulic press at *p* = 3 bar. Activation of the sample was carried out at 200 ^o^C. The sample was then cooled to 150 ^o^C and contacted with pyridine vapours. After 10 min the physisorbed molecules were removed by desorption at the same temperature. Spectrum was recorded at 150 ^o^C. Measurements were made from 4000 to 600 cm^−1^ and 100 scans were taken at a resolution of 2 cm^−1^. An MCT detector was used for detection. The acid group concentrations were calculated from the intensity of the Py bands associated with the Brönsted (1544 cm^−1^, PyH^+^) and Lewis (1446 cm^−1^, PyL) centres and the values of the absorption coefficients of 0.07 cm/µmol and 0.10 cm/µmol, respectively^30^. The chemical composition of the Zr modified samples was determined by wavelength-dispersive X-ray fluorescence (WDXRF; Spectrometer Axios mAX; Rh lamp). Qualitative spectral analysis was performed using Malvern Panalytical’s SuperQManager software. Sample preparation: Cereox binder (C_38_H_76_N_2_O_2_). Sample : binder ratio = 1 : 3. Homogenisation time approx. 1 min. FTIR DRIFT spectra of monoliths were recorded using Nicolet 6700 (Thermo Scientific) device with a resolution of 4 cm^−1^ to identify hydroxyls on the silica surface.

### Activity studies

Continuous-flow experiments were carried out at 70 ^o^C with a flow rate of 0.03 ml/min using a syringe pump. The reaction mixture composed of benzaldehyde dimethyl acetal (10 mmol), ethyl cyanoacetate (10 mmol), water (10 mmol) and 50 ml of solvent (toluene, acetonitrile or toluene/acetonitrile volume ratio of 4/1). For the first 60 min, the reactor was conditioned by passing the solution through the reactor. Samples were collected every 30 min and substrate and product concentrations were analysed by gas chromatography (Agilent, HP-5 column, FID detector). Batch process was carried out at 70 ^o^C under vigorous stirring using 10.8 ml of the reaction mixture and 0.15 g of powdered modified monolith.

## Results and discussion

### Characterisation of monolithic cores

**Fig. 1:**
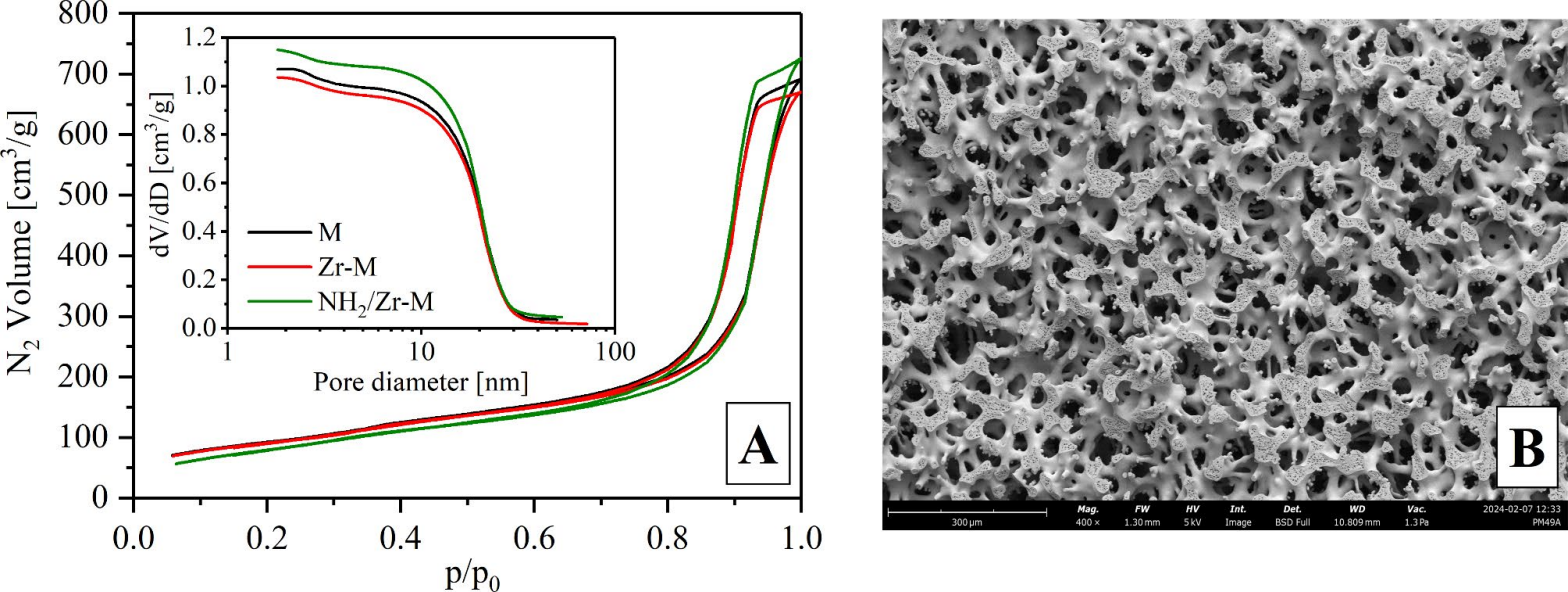
Nitrogen adsorption/desorption isotherms and cumulative pore size distribution for silica monolith and after modification with zirconia and diamine groups (**A**); SEM image of silica monolith (**B**).

Bifunctional microreactors were fabricated using silica monolith as a support for acid-base catalysts. The pores of the monoliths occur at two different size scales, therefore, SEM and nitrogen adsorption techniques were used to determine structure parameters and evaluate the effect of the double modification. The nitrogen adsorption-desorption isotherms display a IV type and H1 hysteresis with a sharp increase in N_2_ volume at p/p_0_ in the range of 0.7–0.9 (Fig. 1A). The two dominant mesopore sizes i.e. ~2.5 nm and 20 nm can be seen in Fig. 1A (inset). The SEM images confirmed the presence of large interconnected flow-through pores with dimensions of 20–60 μm, forming an open channel system throughout the monolith (Fig. 1B). The functionalisation of silica hardly affected the mesostructure. Only a slight reduction of the specific surface area in the monolith was observed. The double modification of the material also had no effect on the parameters of macroporous structure (Supplementary Table S1).

Figure S1(see Supplementary Materials) shows the thermal analysis data for the amine functionalised Zr-M monolith. The initial weight loss of 2% below 150 °C corresponds to the desorption of water from the material. The decomposition of functional groups was observed in the range of 180–700 °C. In this process, not only organic amino groups were released, but also ethyl groups, which were attached to the silica surface during grafting as a result of the esterification reaction of ethanol with surface hydroxyls, as previously discussed^31^. The DTG curve clearly showed the well-developed peaks with maximums at 280 °C and 350 °C, which indicated that thermal decomposition of the aminoethylaminopropyl group occurred in two steps. The total weight loss was approximately 2.4%, corresponding to a concentration of attached ligands of 0.23 mmol/g and a surface coverage of amine groups (NH and NH_2_) equal to 0.86 nm^−2^.

The number of acid sites was determined from pyridine adsorption (Supplementary Fig. S2). The concentration of Brönsted and Lewis centres, involved in the deacetalization reaction, for NH_2_/Zr-M monoliths, was 4 µmol/g and 76 µmol/g, respectively.

### Active sites distribution

**Fig. 2:**
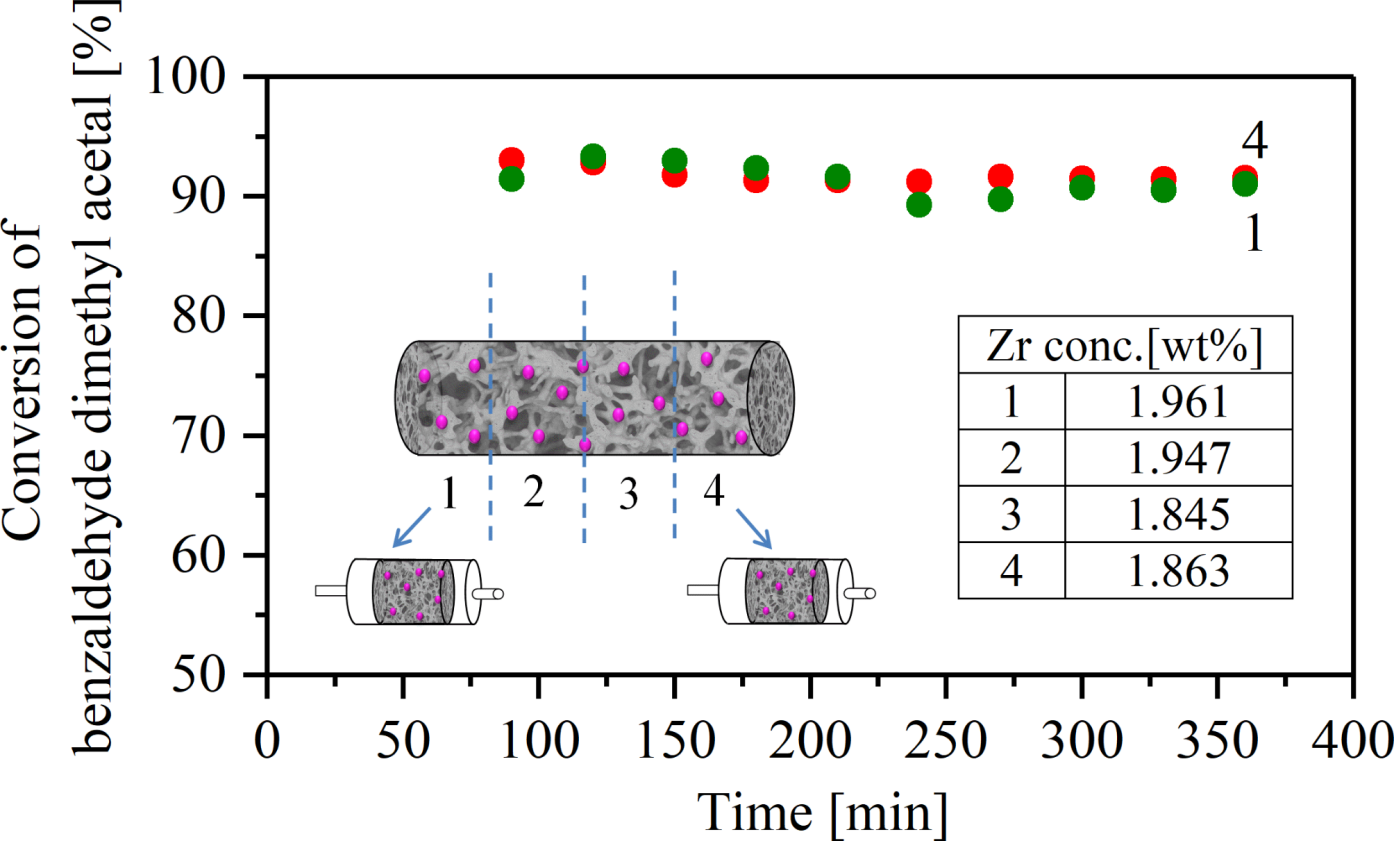
Conversion of benzaldehyde dimethyl acatal in 1 cm-long microreactors. Deacetalization reaction conditions: 70 ^o^C; solvent: toluene/acetonitrile (4/1 v/v). Zr concentration (inset).

The design of bifunctional catalysts for tandem processes requires a uniform distribution of each type of active site on the surface. This is particularly important in the case of moieties with antagonistic behaviour. The aim is to keep the centres at such a distance that they do not interfere with each other but close enough to be able to cooperate. In the case of the monolith, this also refers to an even distribution of catalytic centres along the cylindrical material. The distribution of zirconium was verified in a catalytic test and by elemental analysis using the XRF method. The zirconia functionalised monolith was divided into four parts, each 1 cm long, and they were used as microreactor cores. In all reactors, almost the same conversion of benzaldehyde dimethyl acetal was obtained in the deacetalization reaction, which confirmed the uniform distribution of Zr along the monolith. Also the XRF analysis proved a fairly uniform distribution of zirconium (Fig. 2 inset). The zirconium concentrations in each of the four pieces differed by a maximum of 5.9%.

**Fig. 3:**
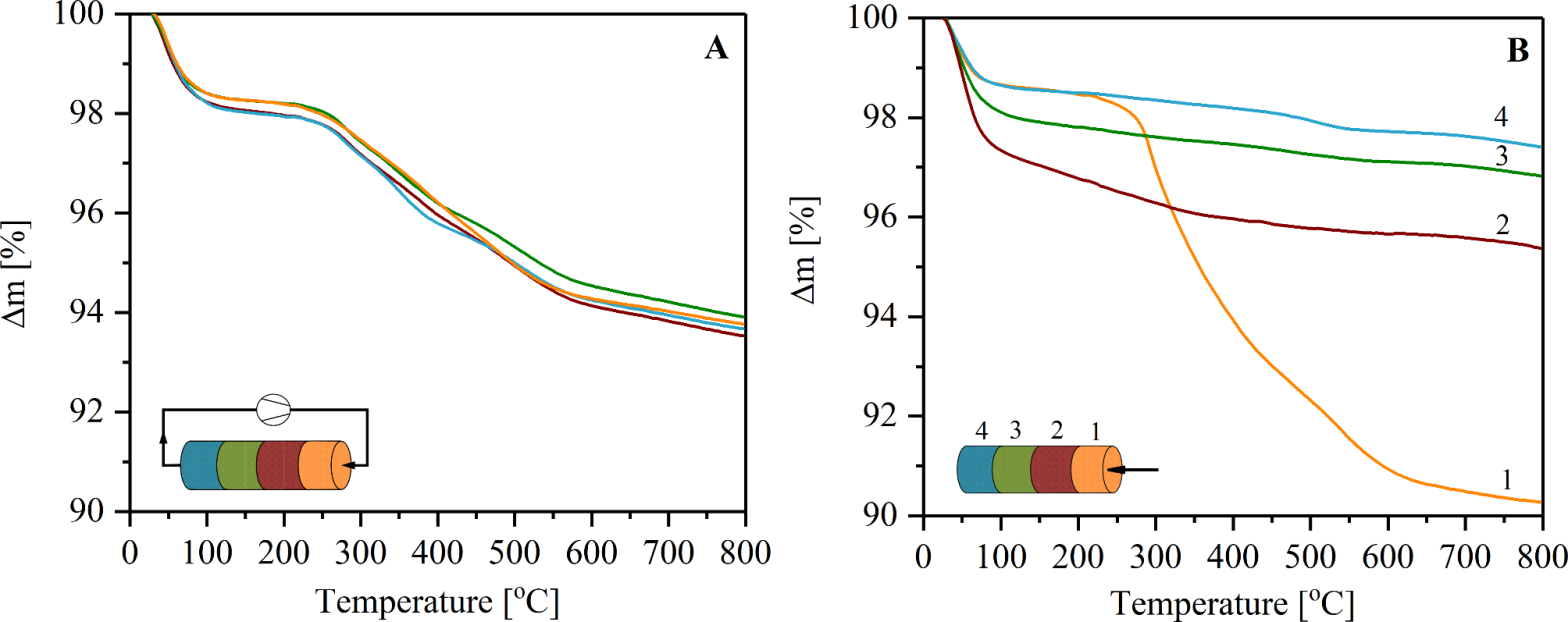
TG curves for amine functionalized monoliths using flow (**A**) and soaking (**B**) methods.

The diaminopropyl ligands were attached to the zirconia/silica surface using flow and soaking methods (see Experimental Section). After modification, the monoliths were cut into four slices and analysed using thermogravimetry (Fig. 3) to compare the amount of functional groups attached.

The total amount of diamine groups in A and B monolith was 0.23 mmol/g and 0.20 mmol/g, respectively. The mass loss in all four parts was almost equal in the monolith modified in the flow (Fig. 3A), which confirmed a homogeneous distribution of the base sites along this monolith. The application of soaking method resulted in a material with a clearly visible concentration gradient (Fig. 3B). The 67% of the introduced ligands were attached in the first part, from the solution inlet (pointed by an arrow).

Both active centres were attached to the silica surface using the grafting method, i.e. by the condensation reaction of surface hydroxyls with zirconium propoxide and 3-(2-aminoethylamino)propyldimethoxymethylsilane. The silica monoliths prior to modification were calcined at 500 ^o^C, and the surface concentration of the hydroxyls, determined using the method proposed in^32^, was 1.8 OH/nm^2^. The surface coverage of the Zr and NH_2_-C_2_H_4_-NH-C_3_H_7_- groups was equal to 0.40 Zr atom/nm^2^ and 0.43 group/nm^2^, respectively. The amine group precursor has two methoxy groups that can react with one or two hydroxyls, while zirconium propoxide, although it has four alkoxy groups, it can react at most with two OH groups^33^, thus there were enough hydroxyls on the silica surface to achieve a uniform distribution of active centres. Moreover, FTIR analysis of silica monolith after functionalization with Zr and then with amine was performed (Supplementary Fig. S3) and it clearly showed a decrease in intensity of the characteristic absorption band for isolated hydroxyls at 3748 cm^−1^. It confirms the attachment of Zr and amine groups to the silica surface, as well as the fact that a small amount of hydroxyls still remains on the surface. Visual representation of the active centres is shown in Fig. S4.

### Catalytic results

**Fig. 4:**
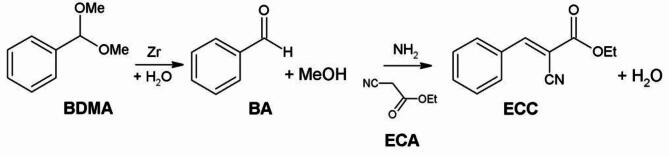
Tandem reaction pathway.

The bifunctional NH_2_/Zr-M reactors were tested in the tandem deacetalization-Knoevenagel condensation reaction. In this process, benzaldehyde (BA) obtained in hydrolysis of acetal (BDMA), reacts directly with ethyl cyanoacetate (ECA) to produce ethyl α-cyanocinnamate (ECC) (Fig. 4).

**Fig. 5:**
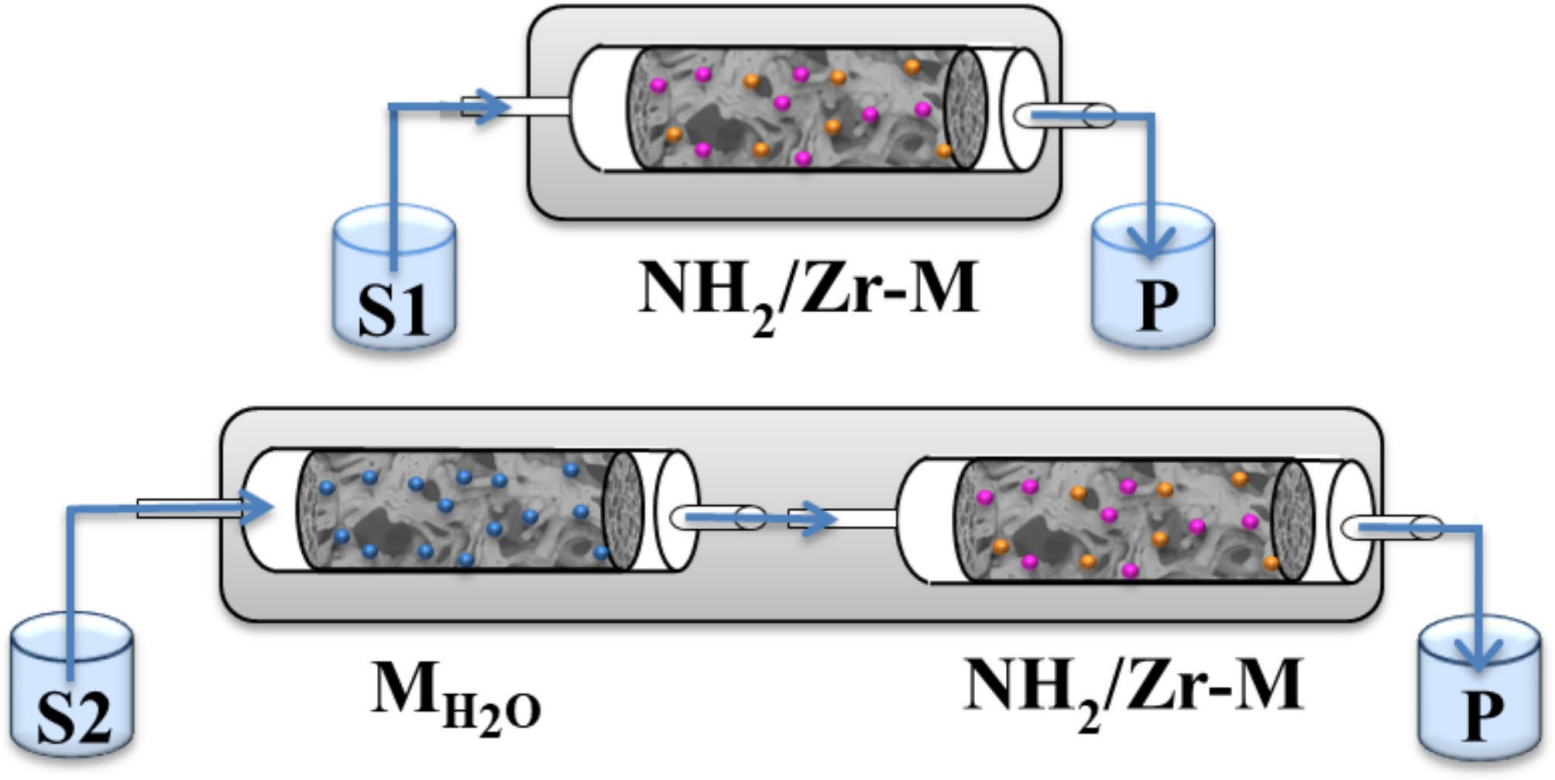
Scheme of NH_2_/Zr-M and ${\text M}_{{\text H_{2}}\text O}$/Zr-M systems. Solution S1: toluene, ACN, H_2_O, BDMA, ECA. Solution S2: toluene, BDMA, ECA. P: products.

The selection of a solvent in a tandem process, where the reactions follow different mechanisms, is a challenge. The deacetalization reaction requires a solvent that forms a homogeneous mixture with H_2_O, while the Knoevenagel condensation is promoted by non-polar solvent^31^. The solubility of water in non-polar solvents is negligible, and therefore, the substrate-solvent system had to be modified. Two approaches have been proposed for the efficient supply of water to the reaction flow system. The first concept was the use of toluene and polar aprotic acetonitrile as a co-solvent, which forms a homogeneous ternary mixture with water, while the latter involved the extension of the reaction system with an additional segment, i.e. a silica monolith with adsorbed water (${\text M}_{{\text H_{2}}\text O}$) (Fig. 5). These approaches were designated as NH_2_/Zr-M and ${\text M}_{{\text H_{2}}\text O}$/Zr-M, respectively.

First, the impact of acetonitrile on Knoevenagel condensation was checked in the NH_2_-M microreactor. Fig. S5 (see the Supplementary Materials) showed that a constant conversion of 90% and 100% selectivity was achieved using both toluene and its mixture with ACN. Under the same conditions, the use of sole ACN resulted in a rapid decrease in catalyst activity over 6 h.

**Fig. 6:**
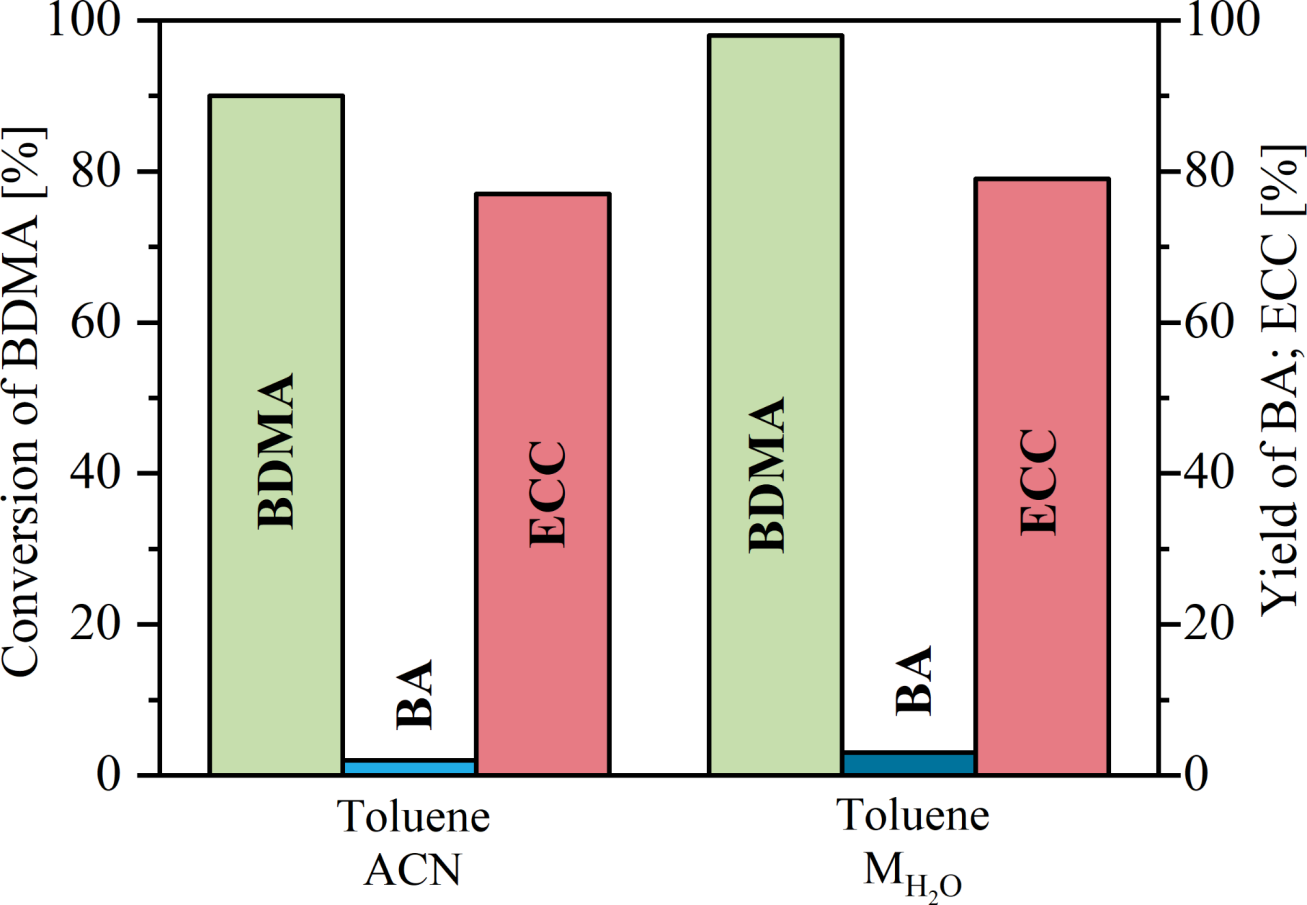
Conversion of benzaldehyde dimethyl acetal and yield of ethyl α-cyanocinnamate and benzaldehyde in tandem deacetalization-Knoevenagel reaction performed in NH_2_/Zr-M and ${\text M}_{{\text H_{2}}\text O}$/Zr-M microreactors.

The performance of the tandem reaction was shown in Fig. 6. The bars represent the average conversion/yield obtained in the microreactors over 6 h. Experiments were carried out for a residence time of 20 min (flow rate of 0.03 ml/min). In both systems, the acetal conversion was greater than 90% and only a small amount of unreacted benzaldehyde remained. Product yields of 77% and 79% were obtained for the NH_2_/Zr-M and ${\text M}_{{\text H_{2}}\text O}$/Zr-M systems, respectively. In both cases, a by-product was detected by GC analysis and identified as methyl α-cyanocinnamate (MCC). It appeared that the methyl alcohol produced in the deacetalization of the benzaldehyde dimethyl acetal undergoes transesterification with nitrile ester (ECA) to give a methyl derivative of the cyanoacetate intermediate (MCA), which further reacted with benzaldehyde (Supplementary Fig. S6).

The impact of temperature on conversion and yield in the studied tandem reaction was performed for the ${\text M}_{{\text H_{2}}\text O}$/Zr-M system, and it has been found that increasing the temperature by 10 ^o^C resulted in higher yield of ECC (95%) and selectivity ~ 100% (Supplementary Fig. S7).

To verify the effect of double modification on the particular process steps, the deacetalization and the Knoevenagel reaction were performed separately in the NH_2_/Zr-M microreactor and additionally in those modified with single catalyst, i.e. zirconium oxide (Zr-M) or amines (NH_2_-M) and compared with the tandem process. Table 1summarizes the results of these experiments. In the deacetalization reaction, the conversion of acetal was about 10% lower in the bifunctional reactor (entry 1) than in that doped with zirconia only (entry 2), which may suggest that some of the acid groups were blocked during the functionalization of Zr-M monolith with amine group precursor or because of the presence of methyl group attached to the surface during amine functionalization. To check this, a microreactor functionalized with 3-(2-aminoethylamino)propyltrimethoxysilane precursor (without methyl group) was fabricated and tested (entry 10). The conversion of BDMA was significantly higher (98%), which confirmed the negative effect of the methyl group on the course of the hydrolysis reaction. The hydrophobic nature of this group can reduce the accessibility of water molecules to acid centres in the first reaction. However, the lack of hydrophobic environment has an impact on the efficiency of the condensation reaction, resulting in a lower yield of ECC (68%) in agreement with our previous studies^31^. The negative impact of neighbouring groups is hardly visible in the Knoevenagel reaction (entry 4 vs. 5), which confirmed that there is no interaction between Zr sites located on the silica surface and the amine groups attached via the propyl linker. In the tandem reaction (entry 7) the conversion of BDMA was 90%, while during deacetalization reaction performed in the bifunctional microreactor (entry 1) it reached a lower value (80%). The higher conversion was probably because of a shift in the equilibrium of the deacetalization reaction, which resulted from the formation of water in the condensation reaction. The yield of the ECC product in the tandem reaction was lower than in the single condensation reaction. It was the effect of the formation of the by-product in the transesterification reaction on the acid catalyst, which was explained earlier. A low conversion of acetal (ca. 8%) to benzaldehyde was detected in the deacetalization reaction carried out in the amine-functionalized microreactor. This can be assigned to the presence of hydroxyl groups on the monolith surface, which exhibit weak acidic properties. The presence of these hydroxyls was confirmed by FTIR analysis (see the Supplementary Fig. S3).

Catalytic experiments were performed in batch reactor to determine an initial rate of single reaction using bi-functionalized catalyst. The initial rate of the deacetalization reaction was found to be 0.11 mol∙h^−1^∙g^−1^, and 0.03 mol∙h^−1^∙g^−1^ for the Knoevenagel condensation. It indicates that the condensation reaction determines the overall rate of the tandem process.

The long-term process was performed in 5 cycles, each for 6 h, and it showed that the deacetalization reaction occurred with the same efficiency, while a decrease in ECC yield was observed from third cycle (Supplementary Fig. S8). The same relationship was found in our previous studies conducted for the condensation reaction in microreactors functionalized with amine groups^31^, and also by other researchers^34^. These results confirmed that the acid centres remain their catalytic properties, while the base sites lose slowly catalytic activity, probably by poisoning^35^.

**Table 1:** Summary of catalytic results for mono- and bifunctional microreactors obtained in deacetalization, Knoevenagel condensation and tandem reactions.

Entry	Reaction	Microreactor	Conversion of BDMA [%]	Yield of BA [%]	Isolated yield of ECC [%]	Selectivity [%]
1	DEACETALIZATION	NH_2_/Zr-M	80	80	-	-
2		Zr-M	93	93	-	-
3		NH_2_-M	8	8	-	-
4	CONDENSATION	NH_2_/Zr-M	-	-	92	100
5		NH_2_-M	-		90	100
6		Zr-M	-	-	-	-
7	TANDEM	NH_2_/Zr-M	90	2	77	88
8		Zr-M	89	89	0	0
9		NH_2_-M	4	1	3	100
10		NH_2_/Zr-M^*^	98	1	68	70

Reaction conditions: 70^o^ C, solvent: tolenene+ACN (4/1 v/v) ; * [3−(2−aminoethylamino)propyl] trimethoxysilane precursor.

**Fig. 7:**
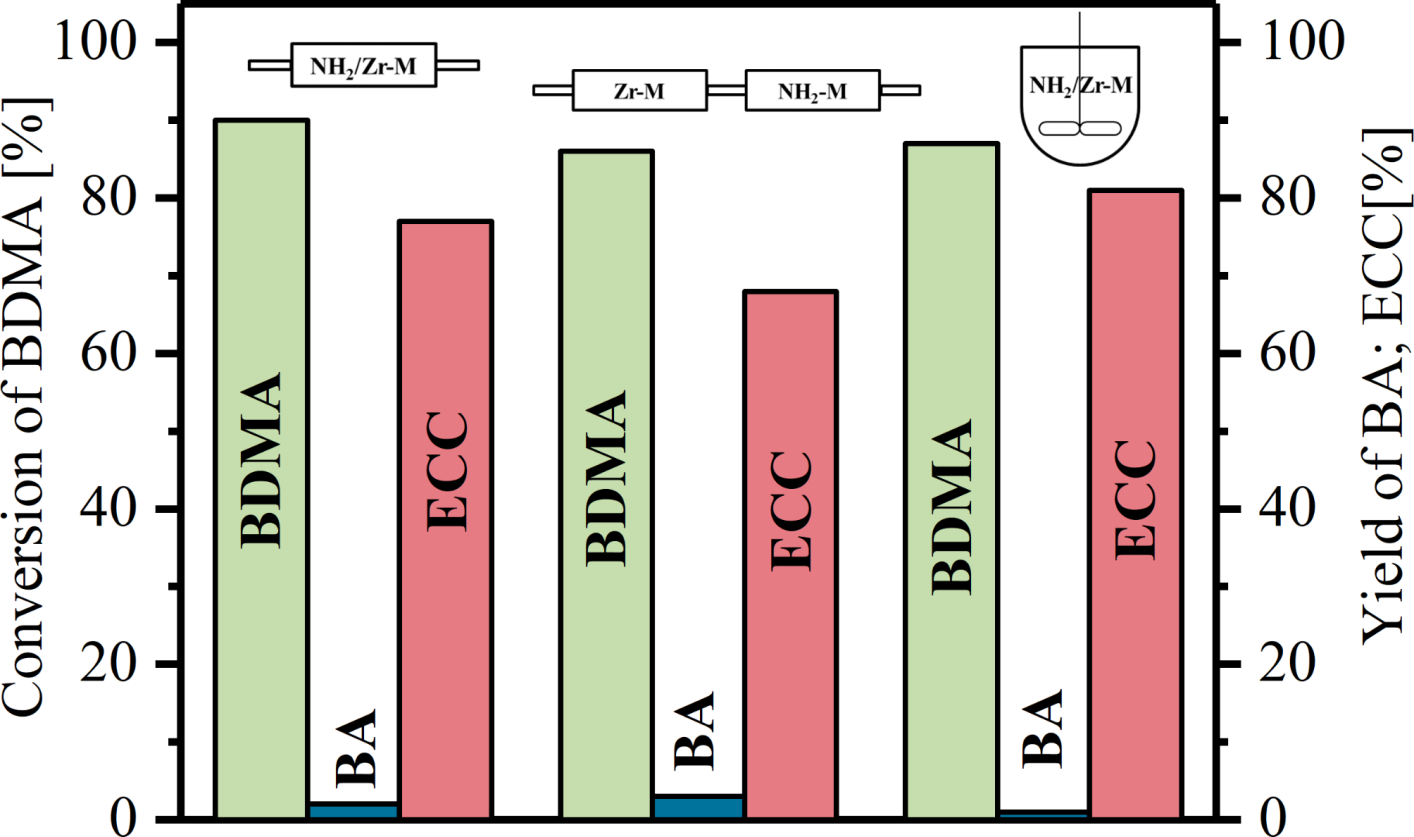
Comparison of the effectiveness of various reactors in tandem process.

Finally, the performance of the bifunctional reactor was compared with the monofunctionalised reactors connected in cascade and also with the batch reactor (Fig. 7). The length of each connected reactor was 4 cm, in order to keep the same residence time of the reactants for both reactions. In this system, a decrease (ca. 10%) in the yield of ECC was observed compared to the bifunctional reactor. Here, at the 1st stage the ethyl cyanoacetate present in the feed mixture has more favourable conditions for reacting with the methanol formed in the deacetalization reaction, and thus the amount of by-product (methyl α-cyanocinnamate) was larger than in the NH_2_/Zr-M reactor. Moreover, in this system twice the pressure drop was recorded. The conversion of acetal was almost the same in all three systems.

In the batch reactor, the amount of catalyst used in the reaction was equal to the mass of the bifunctional microreactor, and the volume of solution was equal to that passed through the microreactor during 6 h of operation. The powdered monolith maintained its meso-scale structural properties. To achieve a conversion close to that in the microreactor, the process should be performed at least 5 h, while in the continuous system first portions of solution with final composition are obtained after only about 20 min, which results from the residence time in the reactor. The higher efficiency of the ECC was observed because of the formation of a smaller amount of by-product, as a consequence of the partial evaporation of methanol from the reaction mixture into the reactor space, despite the presence of a reflux condenser. Nevertheless, the batch process requires additional operations, such as separation of the catalyst by filtration, which is not an issue in flow processes.

## Conclusions

Silica monoliths with hierarchical meso- and macropore structure were functionalized with zirconia and aminoethylaminopropyl groups and used as the core of a microreactor. The performance of the elaborated micro device was verified in the tandem deacetalization-Knoevenagel condensation process, which requires bifunctional acid-base catalyst. The high surface area of the pristine monolith ensured a good dispersion of zirconia along the monolith, which was evidenced by elemental analysis and a simple reaction test. The method of circulating a diamine precursor solution through zirconia-modified monoliths appeared to be more efficient for the uniform attachment of active organic groups than filling the support by capillary forces, as confirmed by TG analysis. Two strategies were proposed for the effective supply of water to the tandem process, in which a hydrophobic organic solvent was necessary to achieve a high yield of the condensation product, i.e. ethyl cyanocinnamate, and it was obtained in both cases. Research carried out in tandem and single reactions using bi- and monofunctionalised microreactors indicated a significant impact of hydrophobic properties of the amine group precursor on the course of hydrolysis and condensation reaction. No effect of zirconia on the activity of the amine groups was observed. It was found that the condensation reaction determines the overall tandem reaction rate. The performance of the flow-through bifunctional microreactor, a cascade of monofunctional reactors and batch reactor were compared, and the advantages of using the bifunctional microreactor were clearly demonstrated. Long-term experiments showed stability of acid sites and slow deactivation of amine groups.

## Electronic supplementary material

Below is the link to the electronic supplementary material.

Supplementary Material 1

## Data Availability

The datasets generated during and/or analysed during the current study are available in the RepOD repository 10.18150/ICLHSU.
