# Supplementary material for: Bifunctionalised acid-base hierarchically structured monolithic microreactor for continuous-flow tandem catalytic process of cyanocinnamate synthesis
Source: Sci Rep. 2024 Oct 25;14:25332. doi: 10.1038/s41598-024-77146-7 (PMC11511982; doi:10.1038/s41598-024-77146-7)
Supplement: Supplementary file 1 — Supplementary Material 1 [file 41598_2024_77146_MOESM1_ESM.pdf]

SUPPLEMENTARY MATERIALS

Bifunctionalised acid-base hierarchically structured monolithic microreactor for continuous-flow tandem catalytic process of cyanocinnamate synthesis

Agnieszka Ciemięga*, Katarzyna Maresz, Julita Mrowiec-Białoń

Monolith	S_{BET} [m^2/g]	V_{meso} [cm^3/g]	d_{meso} [nm]	V_{total} [cm^3/g]	d_{macro} [μm]
M	322	1.07	2.5/20	4.1	20-60
Zr-M	319	1.02	2.5/20.5	4.1	20-60
NH_2/Zr-M	307	1.08	2.5/21	4.1	20-60

Table S1. Structural parameters of materials.

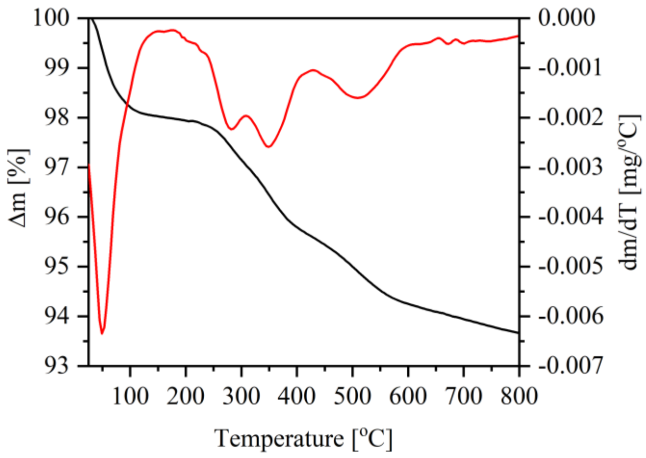

Figure S1. TG and DTG curves for NH_2/Zr-M sample.

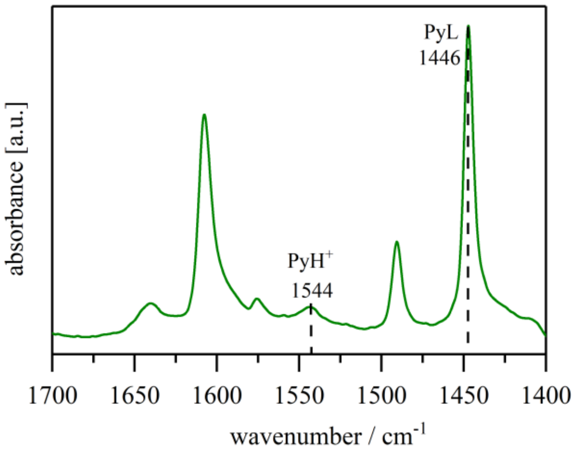

Figure S2. FTIR spectrum of NH_2/Zr-M monolith after pyridine adsorption at 150 ^{o}C.

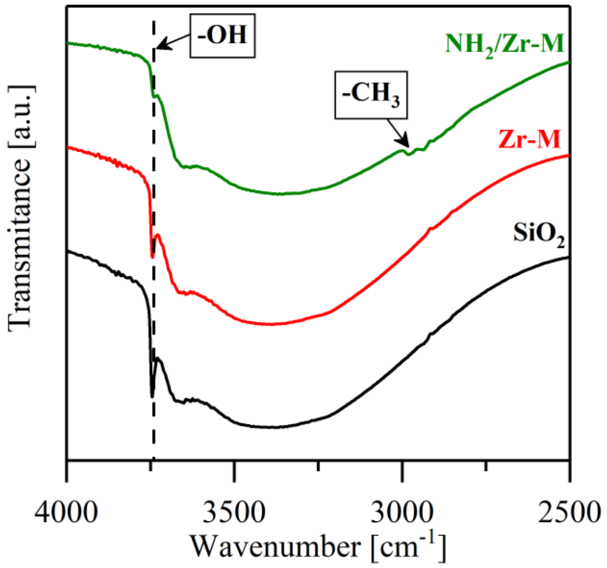

Figure S3. FTIR spectra of silica, Zr-M and NH_2/Zr-M monolith.

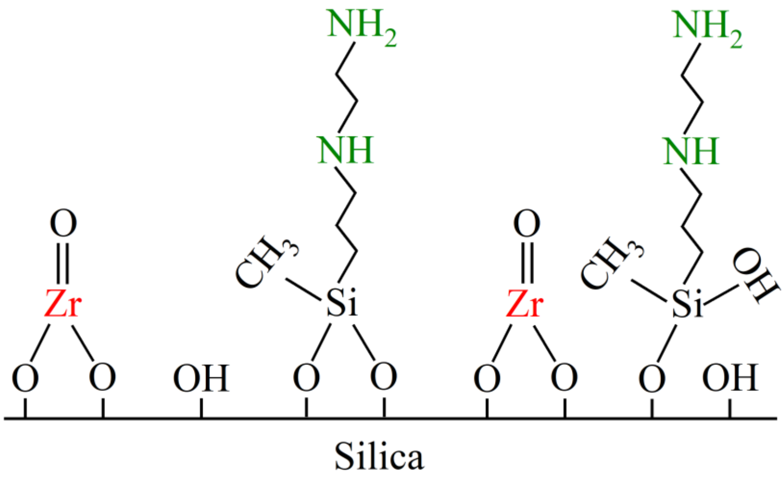

Figure S4. Visual representation of active centres of NH_2/Zr-M monolith.

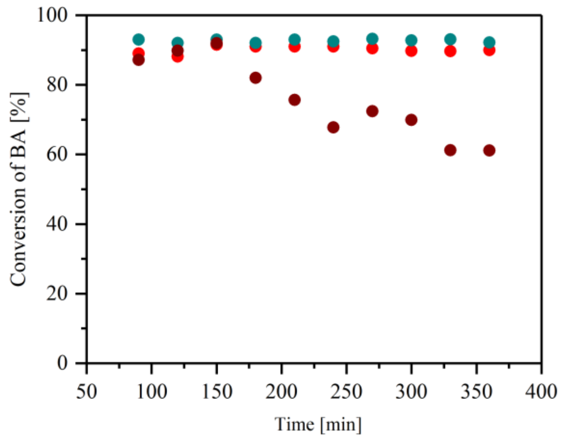

Figure S5. Conversion of benzaldehyde vs. time in Knoevenagel reaction performed in NH_2/M microreactors with toluene, ACN and mixture of both solvents (4/1 v/v).

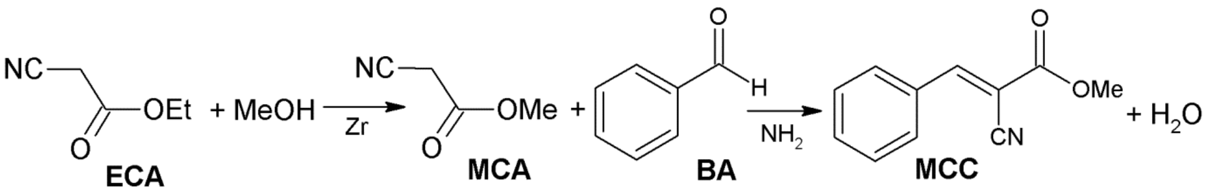

Figure S6. Reaction pathway of by-product synthesis.

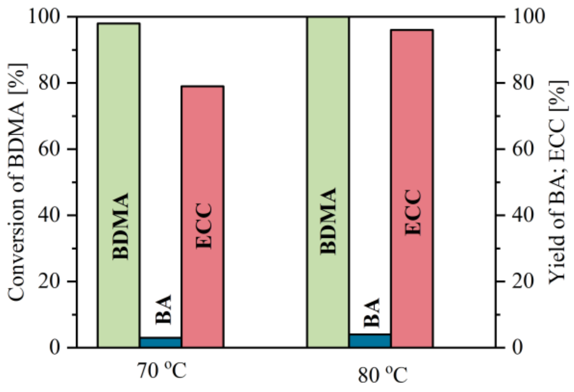

Figure S7. Influence of temperature on conversion and yield for M_{H2O}-NH_2/Zr-M system.

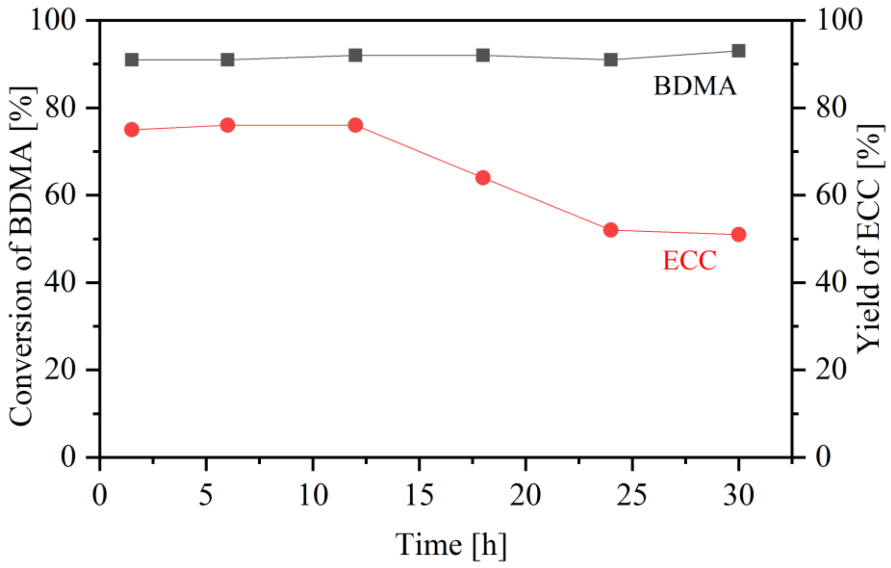

Figure S8. Long-term experiment of tandem process.
